# Study on microstructure and mechanical properties of 3003 aluminum alloy joints brazed with Al–Si–Cu–Ni paste brazing materials

**DOI:** 10.1038/s41598-024-61166-4

**Published:** 2024-05-09

**Authors:** Zeng Gao, Xiwei Jin, Shuaiqi Li, Zhen Zhang, Jitai Niu, Josip Brnic

**Affiliations:** 1 School of Materials Science and Engineering, Henan Polytechnic University, Jiaozuo, 454003 China; 2 State Key Laboratory of Advanced Welding and Joining, Harbin Institute of Technology, Harbin, 150001 China; 3 Faculty of Engineering, University of Rijeka, 51000 Rijeka, Croatia

**Keywords:** Engineering, Materials science

## Abstract

Paste-type brazing materials have advantages such as adjusting the complexity of the parts to be soldered, easy storage and production in certain quantities. They can be used for brazing heat exchangers, liquid tanks and corrosion resistant parts. In this work, the microstructures and thermal behaviors of Al–Si–Cu–Ni brazing materials with different contents were investigated, and the effect of brazing process on the microstructural evolution and mechanical properties of brazed joints produced under nitrogen-filled environment was examined. It was found that the melting temperature of brazing material Al–5Si–20.5Cu–2Ni were ranged from 512.86 to 549.37 °C. The microstructure of Al–5Si–20.5Cu–2Ni consisted of α-Al solid solution, CuAl_2_ intermetallic compounds, the Al–Si–Cu phase, and some fine irregular Si particles in a homogenous manner. The microstructure of the brazed joints was uniformly formed during the brazing condition of 580 °C for 20 min, and the shear strength of the joints reached 41.76 MPa.

## Introduction

3003 antirust aluminum alloys are widely used for parts that work interactively with gaseous and liquid media due to their low density, good processing and corrosion resistance. The commonly used brazing material for 3003 aluminum alloy, Al–Si eutectic^1–4^, poses challenges due to high process temperature, which closely approaches the solid phase line temperature of the 3003 alloy. This proximity in temperatures during brazing processes can lead to base material deformation, melting, and weakened structural integrity^5,6^. In recent years, Chuang et al^7^. reported that the liquid-phase line temperature rises to 572.8 °C when the Cu content in the Al–Si–Cu alloy is increased to 40 percent. Zhang et al^8^. brazed a 6063 aluminum alloy with Al–8.5Si–25Cu–1.5Ni–0.2Re with high Cu content and obtained an average strength of 62.5 MPa. Thus, it is of great practical significance to find a low melting point aluminum-based brazing material^9^ to reduce the brazing temperature and prepare it as a paste brazing materials to achieve low-cost and high-quality brazed joints of 3003 aluminum alloy.

Aluminum-paste brazing materials primarily consist of three components, brazing flux, solvent and brazing material. Thorough stirring is required before use if the paste brazing material shows signs of stratification. The performance of the brazing material significantly influence the quality of the brazed joint^10–13^. In this study, Al–Si–Cu–Ni low melting point brazing materials were prepared into material powders with suitable particle size using nitrogen atomization technology. Material powders prepared on the basis of nitrogen atomization method not only have the characteristics of low melting point brazing materials, but also exhibits advantages as low oxidation, uniform composition and balanced particle size distribution^14,15^.Microstructural observation and mechanical performance testing were conducted on brazed joints using 3003 aluminum alloy as the base material to investigate the impact of brazing processes on joint reliability.

## Materials and experiments

In this study, the base material choose was corrosion-resistant aluminum alloy 3003, an Al-Mn alloy that doesn’t undergo strengthening through heat treatment. The size used for the experiment was 10 mm × 20 mm × 2 mm. The solidus temperature of 3003 aluminum alloy is 643 °C and the liquidus line temperature is 655°C^16,17^.

**Figure 1:**
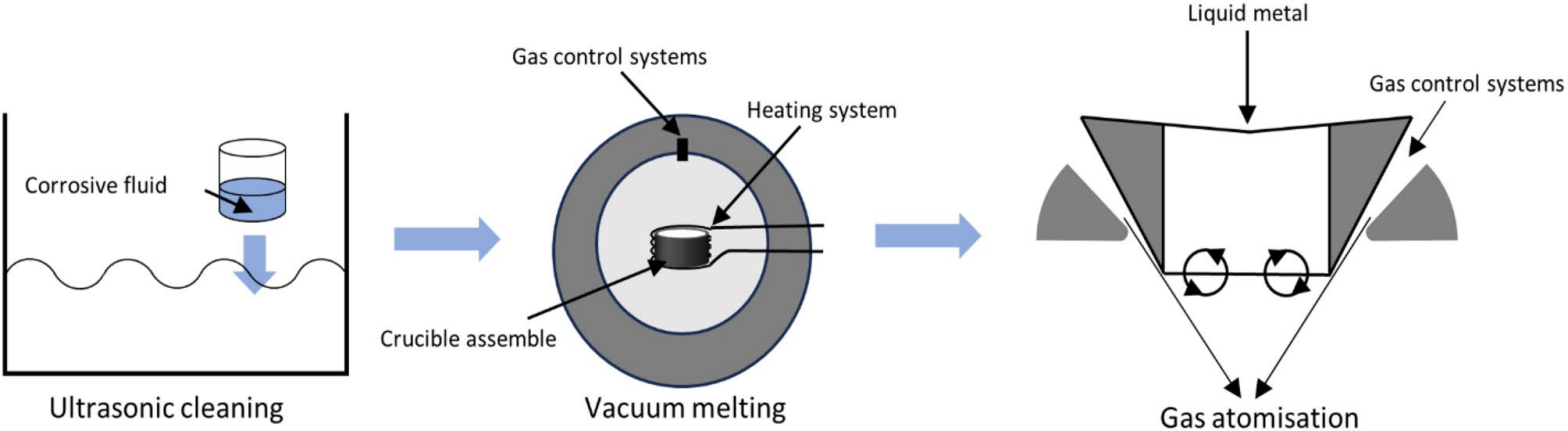
Schematic diagram of brazing alloy preparation process.

The brazing materials used in the study were made from Al, Si, Cu and Ni with high purity. The fabrication process of the brazing material is illustrated in Fig. 1. Initially, the raw materials underwent cleaning in an ultrasonic cleaner using a solution of 5% NaOH, 5% HNO_3_ and acetone alchol.Subsequently, they wrer melted three times in an induction furnace under high purity argon gas. Once the composition of the brazing material was determined, powdered brazing material was produced through gas atomisation in a nitrogen-protected environment. The paste brazing material was synthesised from a combination of potassium chlorofluoride (K_3_AlF_6_) brazing flux, Al–5Si–20.5Cu–2Ni brazing material powder and organic reagents, meticulously mixed in a vacuum glove box. The melting characteristics of the brazing compounds were assessed using Differential Scanning Calorimetry (DSC,STA449F3-QMS403Q), while the microstructure of the materials was examined by Scanning Electron Microscopy (SEM, Merlin Compact) and Energy Dispersive Spectroscopy (EDS,OXFOFD).

**Figure 2:**
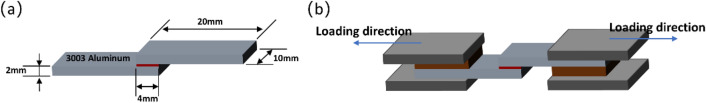
Schematic representation, (**a**) brazing model, (**b**) shearing testing of joints.

The technology parameters were, brazing temperature T = 570–590 °C, holding time t = 10–30 min., Heating was conducted at a rate of 10 °C/min until reaching the target temperature and holding, followed by furnace cooling to room temperature.. The brazing is done with nitrogen protection and lap joints with a lap length of 4 mm. Figure 2 illustrates the geometry and dimensions of the brazed specimens used for mechanical testing and microstructural observation. Mechanical properties were assessed using an electronic universal testing machine (CMT5205, MTS Systems) at a shear rate of 0.5 mm/min. To mitigate test errors, three specimens were tested for each brazing process, and the average shear strength value was calculated.

## Results and discussion

### Thermal behavior and microstructure analysis of brazing alloys

**Figure 3:**
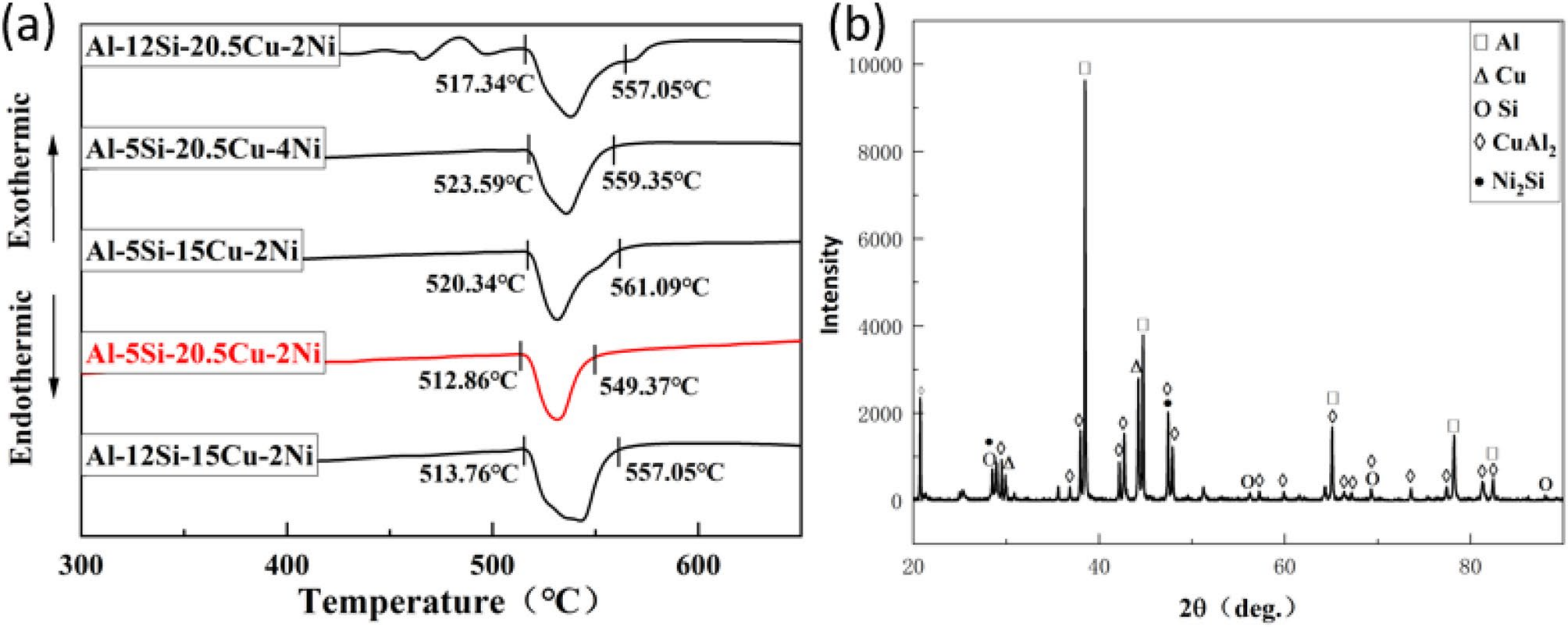
(a) DSC curves of the Al–Si–Cu–Ni filler metals, (b) DSC analysis of Al–5Si–20.5Cu–2Ni filler metals.

Figure 3a presents the results of the DSC analysis for Al–Si–Cu–Ni brazing materials with varying composition. The finding indicates that as the Cu content increases from 15 to 20.5%, the solid-phase line temperature decreases from 520.34 to 512.86 °C, the liquid-phase line temperature decreases from 561.09 to 549.37 °C. Moreover, the peak of heat absorption is shifted to the left. Conversely when the Si content is increases from 5 to 12%, the solid-phase line temperature rises from 512.86 to 517.34 °C. Additionally, the liquid phase line temperature increased from 549.37 to 562.93 °C, and the heat absorption peak shifted to the right. In Fig. 3b, the X-ray diffraction results of the Al-5Si-20.5Cu-2Ni brazing material reveal the presence of α-Al solid solution, CuAl_2_ intermetallic compounds, and some fine irregular Si particles.

**Figure 4:**
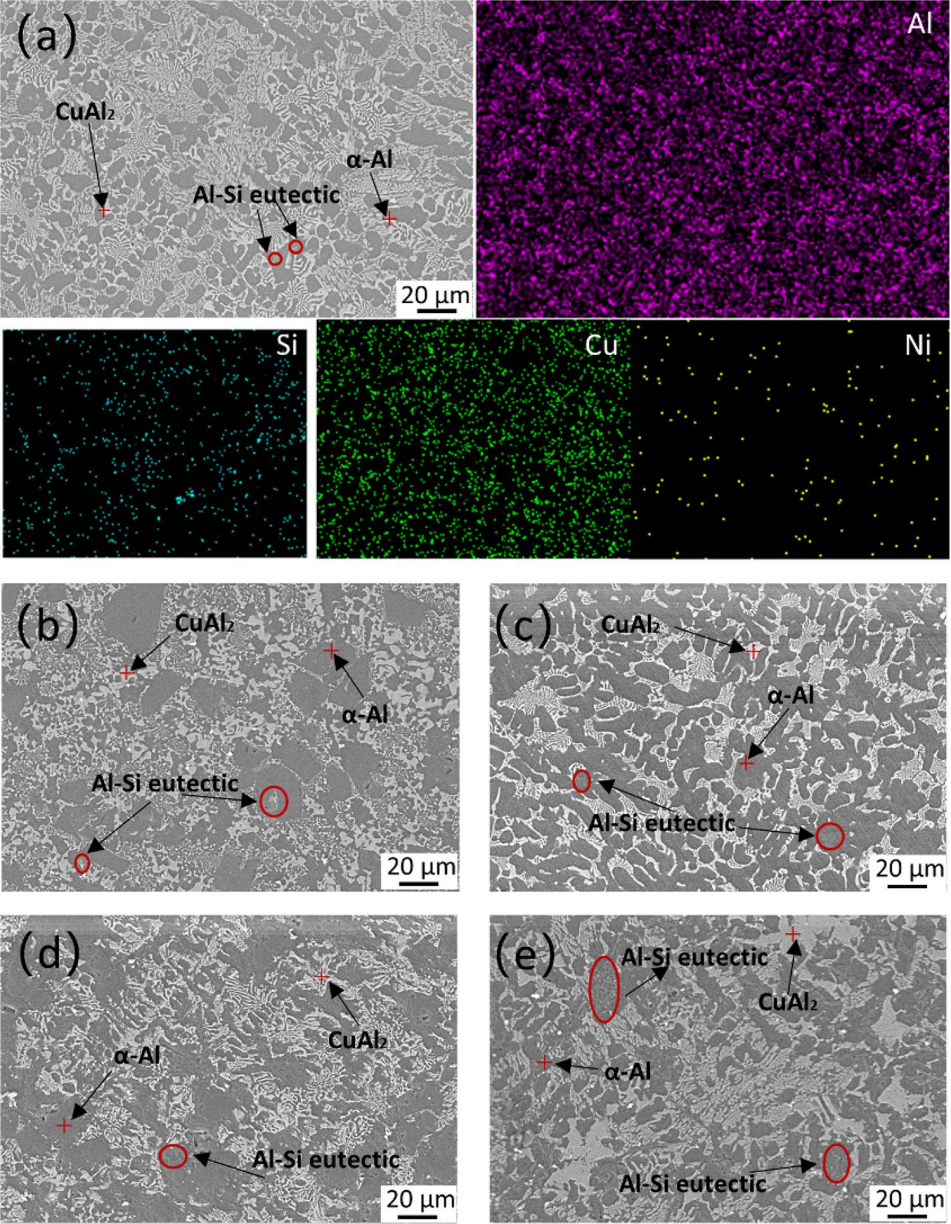
SEM microstructures of (**a**) Al–5Si–20.5Cu–2Ni, (**b**) Al–5Si–15Cu–2Ni, (**c**) Al–5Si–20.5Cu–4Ni, (**d**) Al–12Si–15Cu–2Ni, (**e**) Al–12Si–20.5Cu–2Ni.

From Fig. 4, it is evident that all five brazing materials are composed of dark grey α(Al) solid solution, light grey CuAl_2_ intermetallic compounds, and fine Si particles located between grain boundaries. Specifically, in Fig. 4a, the Al–5Si–20.5Cu–2Ni brazing material exhibits uniform grain size, with the CuAl_2_ intermetallic compounds evenly distributed.

**Figure 5:**
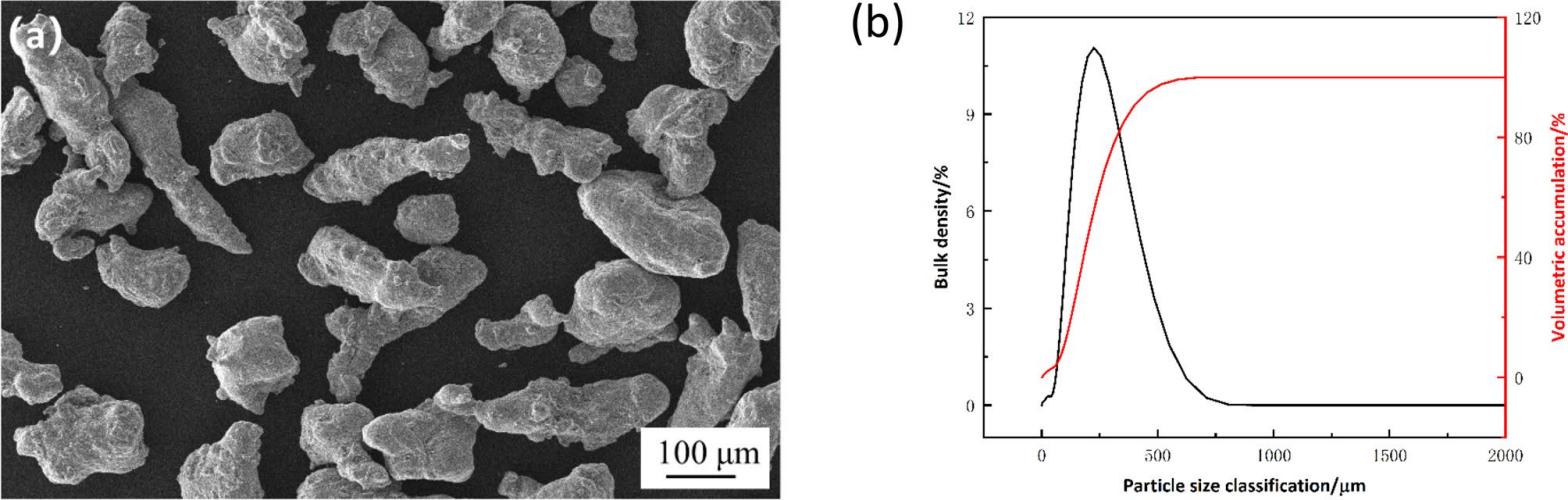
(**a**) SEM micrograph of brazing filler material Al-5Si-20.5Cu-2Ni. (**b**) Particle size distribution of powdered brazing filler material.

The microstructure of the powdered Al-5Si-20.5Cu-2Ni brazing material used in the test is depicted in Fig. 5a, revealing an elliptical shape of the material powder particles. As shown in Fig. 5b, the particle size distribution is predominantly centered around 100 μm. Moreover, the degree of oxidation is low, facilitating good spreading characteristics of the paste brazing material.

### Analysis of microstructure and mechanical properties of brazed joints

**Figure 6:**
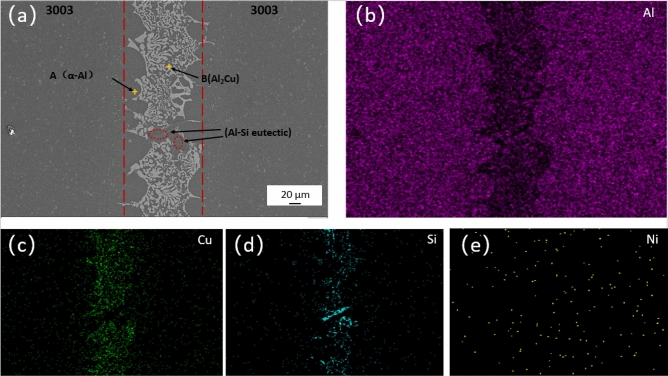
SEM micrograph and EDS analysis of 3003 joint brazed at 580 °C for 20 min.

In Fig. 6, the microstructure of 3003/3003 joint filled with Al–5Si–20.5Cu–2Ni paste brazing material at 580 °C for 20 min is illustrated. In the SEM image (Fig. 6a), the well-bonded brazed joint appears devoid of cracks and fractures. The interfacial zone of the brazed joint comprises a transition zone on the aluminum side and an intermediate weld zone. The point scanning results provided in Table 1 indicate that the generated spherical structure corresponds to α-Al solid solution, while the light gray dendritic structure represents CuAl_2_ compound. Furthermore, in Fig. 6c, it is evident that as the heat process progresses, there is diffusion among the Al element in the base metal and the Al, Si, and Cu elements in the brazing material within the center of the cold weld, leading to the formation of Al–Si eutectic phase. Notably, improper adjustment of the brazing process can hinder the growth of α-Al spherical structures and other intermetallic compounds in the intermediate layer of the brazed joint.

**Table 1:** EDS results of corresponding points in Fig. 6 (at. %).

Position	Al	Cu	Si	Ni	O
A	96.798	1.249	0.585	0	1.367
B	65.3	30.128	1.221	0.647	2.612

**Figure 7:**
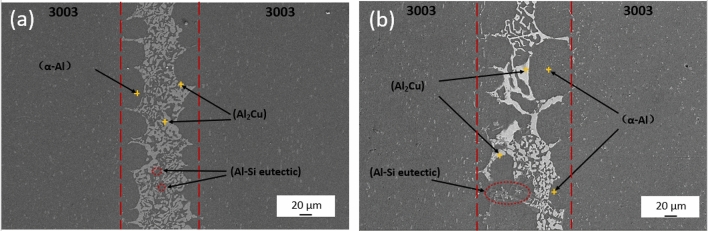
SEM micrograph analysis of joint (**a**) 580 °C, 10 min joint, (**b**) 580 °C, 30 min joint.

It is obvious from Fig. 7a,b that prolonged holding time leads to widening of the weld width. The dark grey spherical structure of α-Al within the diffusion zone tends to detach from the small particles, exhibiting a tendency to separate from the base material. Meanwhile, within the center region of the brazing seam, the fine and dense light grey snowflake-like CuAl_2_ intermetallic compound undergoes gradual growth due to melting, resulting in uneven grain size and distribution. These observed phenomena are attributed to the extension of the holding time, which enhances the elemental diffusion reaction. However, it's important to note that while the mechanical properties of 3003 brazed joints primarily rely on α-Al in the diffusion zone, the presence of a large-sized brittle phase of CuAl_2_ in the central region of the braze seam is detrimental to the mechanical properties of the joints.

**Figure 8:**
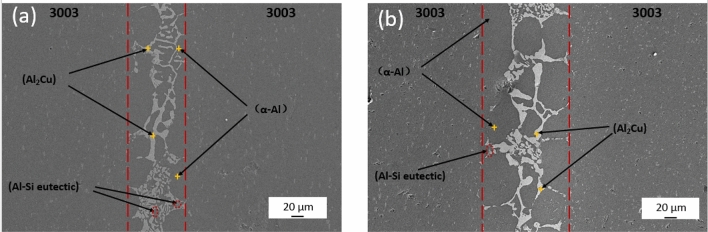
SEM micrograph analysis of joint (**a**) 575 °C, 20 min joint, (**b**) 585 °C, 20 min joint.

The tissue analysis of brazed joints held at different temperatures provides valuable insights into the structural changes during brazing. Brazed joints at 575°C are shown in Fig. 8a, dendritic CuAl_2_ regions decrease in size and homogengity,while coarse needle-like Al–Si transforms into fine spherical particles.At higher temperature, α-Al becomes a separate structure,CuAl_2_ refines due to Cu diffusion, and Al–Si elocates closer to the base material,as seen in Fig. 8b.Comparing the two temperatures,the tissue analysis reveals that deviations from the optimum brazing temperature result in signficant changes in the joint structure,ultimately impacting its mechanical properities.

**Figure 9:**
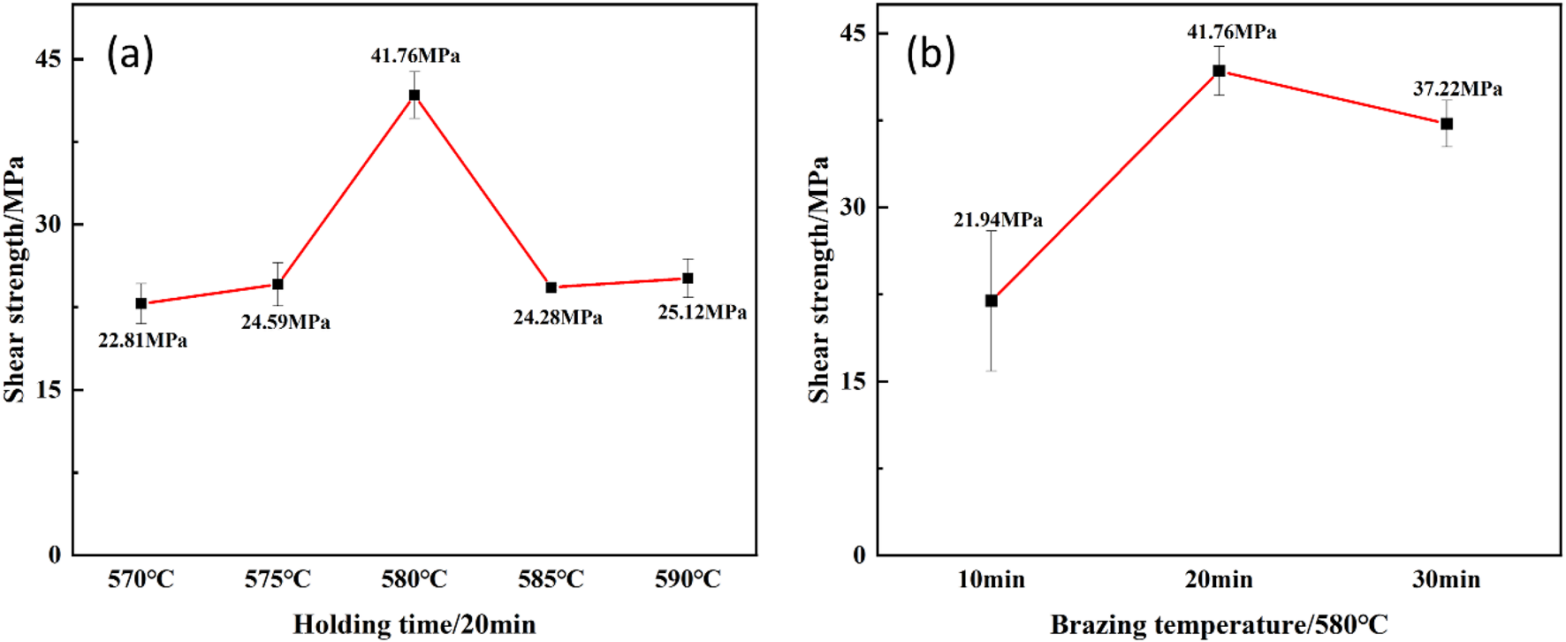
Shear strength under different brazing processes (**a**) Shear strength of different brazing temperature, (**b**) Shear strength of different holding time.

Figure 9 illustrates the shear strength of brazed joints across various brazing processes. With increasing brazing temperature, the joint strength initially rises from 22.81 MPa to a peak of 41.76 MPa and then decreases to 25.12 MPa. The shear strength of the brazed joints reaches a maximum value of 41.76 MPa when the brazing temperature is 580 °C for 20 min. This peak strength is attributed to the presence of well-formed jagged α-Al and dense, fine CuAl_2_ within the joints, optimizing their mechanical properties.

**Figure 10:**
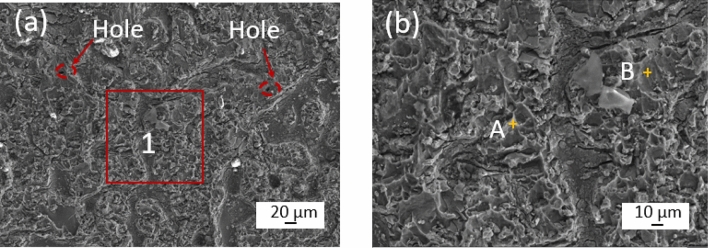
Joint fracture morphology (**a**) aluminum side fracture, (**b**) enlarged view of area 1 in Fig a.

The fracture morphology of the brazed joint is the main basis for analyzing the mechanical properties of the brazed joint. Figure 10 shows the fracture morphology and structure of aluminum base metal after a joint fracture that occurs at the brazing seam. The fracture is relatively flat and plastic deformation during fracture is not obvious. In Fig. 10a, tough nests and tear ribs are observed, indicating that the fracture is a mixed fracture. In order to further investigate the elemental distribution within the fracture zone, energy spectrum scanning analysis was carried out for points A and B in Fig. 10b. As shown in Table 2, the fracture points mainly contain Al and Cu elements. The fracture points are judged to be at α-Al and CuAl_2_ in the brazing seam based on the atomic ratio. When the joint is subjected to external force, the brittle phase is difficult to change the stress state due to its brittleness, which is easy to cause stress concentration and crack initiation. The presence of K and O elements on the fracture surface indicates residues from the brazing flux and solvent. Decreasing these residues within the joint can significantly enhance its performance.

**Table 2:** EDS results of corresponding points in Fig. 10 (at. %).

Position	Al	Cu	Si	Ni	O	K
A	67.3	7.2	2.3	0.3	20.7	2.1
B	35.7	12.7	18.3	0	32.0	1.3

## Conclusion

The obtained Al-5Si-20.5Cu-2Ni brazing material has good thermal properties, with melting temperatures ranging from 512.86 to 549.37 °C and a heat of melting interval of only 36.15 °C, making it suitable for use as a brazing material for 3003 aluminum alloy.

The brazed joints achieved through the utilization of the Al-5Si-20.5Cu-2Ni brazing material primarily comprised spherical α-Al particles of consistent dimensions, interspersed with a light grey dendritic phase of CuAl_2_, and Al–Si eutectic compounds, exhibiting a dense and uniform distribution. The maximum shear strength of the 3003 joints was 41.76 ± 2.12 MPa under the brazing condition of 580 °C for 20 min. The jagged α-Alin the diffusion zone of the brazed joints and the fine and densely distributed CuAl_2_ phase in the center zone are more conducive to the improvement of the mechanical properties of the joints. Fewer residues in the joint and lower brazing temperatures ensure a long life of the joint.

## Data Availability

All data gengerated or analysed during this study are included in this published article.
